# Supplementary material for: Advances in Azorella glabra Wedd. Extract Research: In Vitro Antioxidant Activity, Antiproliferative Effects on Acute Myeloid Leukemia Cells and Bioactive Compound Characterization
Source: Molecules. 2020 Oct 22;25(21):4890. doi: 10.3390/molecules25214890 (PMC7660062; doi:10.3390/molecules25214890)
Supplement: Supplementary file 1 [file molecules-25-04890-s001.pdf]

Supplementary Table S1. Percentage of AML cell viability respect to control used to determine the EC_{50} values of AG samples.

cell line	time	AG sample	CTRL			10 mg/mL			25 mg/mL			50 mg/mL		
KG1	24h	IV	100	100	100	85.91	83.95	83.60	60.06	63.90	63.92	32.42	25.53	30.60
		VI	100	100	100	85.26	79.01	80.00	66.90	64.72	59.53	20.30	24.75	22.12
		AG $CHCl_3$	100	100	100	100	100	96.66	81.73	84.13	83.20	37.74	43.90	40.03
	48h	IV	100	100	100	89.60	77.47	89.60	46.47	41.79	46.20	21.74	23.14	25.66
		VI	100	100	100	85.26	76.01	74.00	64.10	57.72	60.00	17.33	20.12	18.10
		AG $CHCl_3$	100	100	100	97.57	85.91	95.96	90.14	75.78	78.27	22.16	30.31	28.93
MV4-11	24h	IV	100	100	100	54.64	48.83	51.46	49.15	48.11	50.40	39.27	34.66	28.69
		VI	100	100	100	78.32	79.89	74.89	52.00	52.25	51.24	35.55	36.31	26.62
		AG $CHCl_3$	100	100	100	100	100	89.52	99.12	98.52	85.19	49.00	42.08	50.36
	48h	IV	100	100	100	39.00	37.65	31.44	22.51	21.56	20.31	17.63	16.61	21.00
		VI	100	100	100	38.74	53.48	38.13	22.19	23.16	23.70	19.96	18.69	13.51
		AG $CHCl_3$	100	100	100	100	100	100	83.12	100	100	36.44	42.29	37.33
